# Advanced Dental Materials: From Design to Application—Second Edition

**DOI:** 10.3390/ma18214902

**Published:** 2025-10-26

**Authors:** Josip Kranjčić, Tina Poklepović Peričić

**Affiliations:** 1 Department of Prosthodontics, University Hospital Dubrava, Av. Gojka Šuška 6, 10000 Zagreb, Croatia; 2 Department of Fixed Prosthodontics, University of Zagreb School of Dental Medicine, Gunduliceva 5, 10000 Zagreb, Croatia; 3 School of Medicine, University of Split, Šoltanska 2, 21000 Split, Croatia; tinapoklepovic@gmail.com

“Advanced Dental Materials: From Design to Application—Second Edition” is the title of the second edition of a highly successful Special Issue in the journal Materials. When we were initially invited to consider the concept of a Special Issue as guest editors, one of our main ideas was to gather a comprehensive collection of the latest research in the field of dental materials and present it as a unified body of work. That vision has been successfully realized.

Following the unexpected success of the first edition—which culminated in the publication of a printed book under the same title and theme—we made the proud and bold decision to launch a second Special Issue. The dedicated effort and time invested by us, as well as by numerous scientists, clinicians, and esteemed colleagues from around the world (without whom none of this would have been possible), have quickly resulted in another successful volume. This second edition now stands as a coherent and well-defined scientific compilation.

I feel it is important to emphasize the need for the continuous improvement of existing dental materials on the market, as well as the development of new ones. In this context, the role of research in dental materials becomes even more significant, as it not only highlights the outcomes of material design and production but also points to their potential for therapeutic application.

In this spirit, I sincerely hope that this Special Issue will, at least to some extent, contribute to this important field—and, by extension, to the advancement of dentistry as a whole.

This Special Issue features a total of eight high-quality scientific papers published over the course of one year. More than 30 researchers and co-authors from around the world contributed to the publication of their scientific work.

The key themes on which this Special Issue is based include dental materials, ceramics, dental alloys, composite materials, CAD–CAM technology, dental implants, prosthodontics, fixed prosthodontics, removable dental prosthesis base materials, and restorative dentistry.

Matta R.E. et al. (1) contributed to this Special Issue with their article titled “In Vivo Wear Analysis of Leucite-Reinforced Ceramic Inlays/Onlays After 14 Years”; Berger L. et al. (2) contributed with their article titled “Influence of the Milling Strategy on the Marginal Fit of Chairside-Fabricated Lithium Disilicate Crowns”; Strunz A. et al. (3) contributed with their article titled “The Impact of Three-Dimensional Printer Technology on the Accuracy of Dental Implant Models”; Riberti N. et al. (4) contributed with their article titled “Biconometric Connections in Dental Implants: A Pilot Mechanical Study”; Fratila CR. et al. (5) contributed with their article titled “Accuracy Evaluation of Indirect Bonding Techniques for Clear Aligner Attachments Using 3D-Printed Models: An In Silico and Physical Model-Based Study”; Herrero-Climent M. et al. (6) contributed with their article titled “Mechanical Behavior of PEEK and PMMA Graphene and Ti6Al4V Implant-Supported Frameworks: In Silico Study”; Ceddia M. et al. (7) contributed with their article titled “Biomechanical Finite Element Analysis of Two Types of Short-Angled Implants Across Various Bone Classifications”; Murillo-Gómez et al. (8) contributed with their article titled “Mechanical, Adhesive and Surface Properties of a Zirconia-Reinforced Lithium Silicate CAD/CAM Ceramic Exposed to Different Etching Protocols”. The research findings are presented through a series of tables, graphical illustrations, and images, which further enhance the readability and visual appeal of the articles.

This Special Issue—like the one before it—is rich in new scientific insights and serves as a vital link between research and clinical practice. In this way, the studies conducted and the results published herein gain their full meaning and relevance.

Once again, we extend our sincere gratitude to all contributors for their valuable participation in the creation of this Special Issue. Through their dedicated work, they have helped bring this project to life.

